# Supplementary material for: The causal role of circulating inflammatory markers in osteoporosis: a bidirectional Mendelian randomized study
Source: Front Immunol. 2024 Jul 18;15:1412298. doi: 10.3389/fimmu.2024.1412298 (PMC11291241; doi:10.3389/fimmu.2024.1412298)
Supplement: Supplementary file 1 [file DataSheet_1.zip › supplementary material/OSTEOPOROSIS-CIM/GCST90274765.tsv.gz_leave_one_out.pdf]

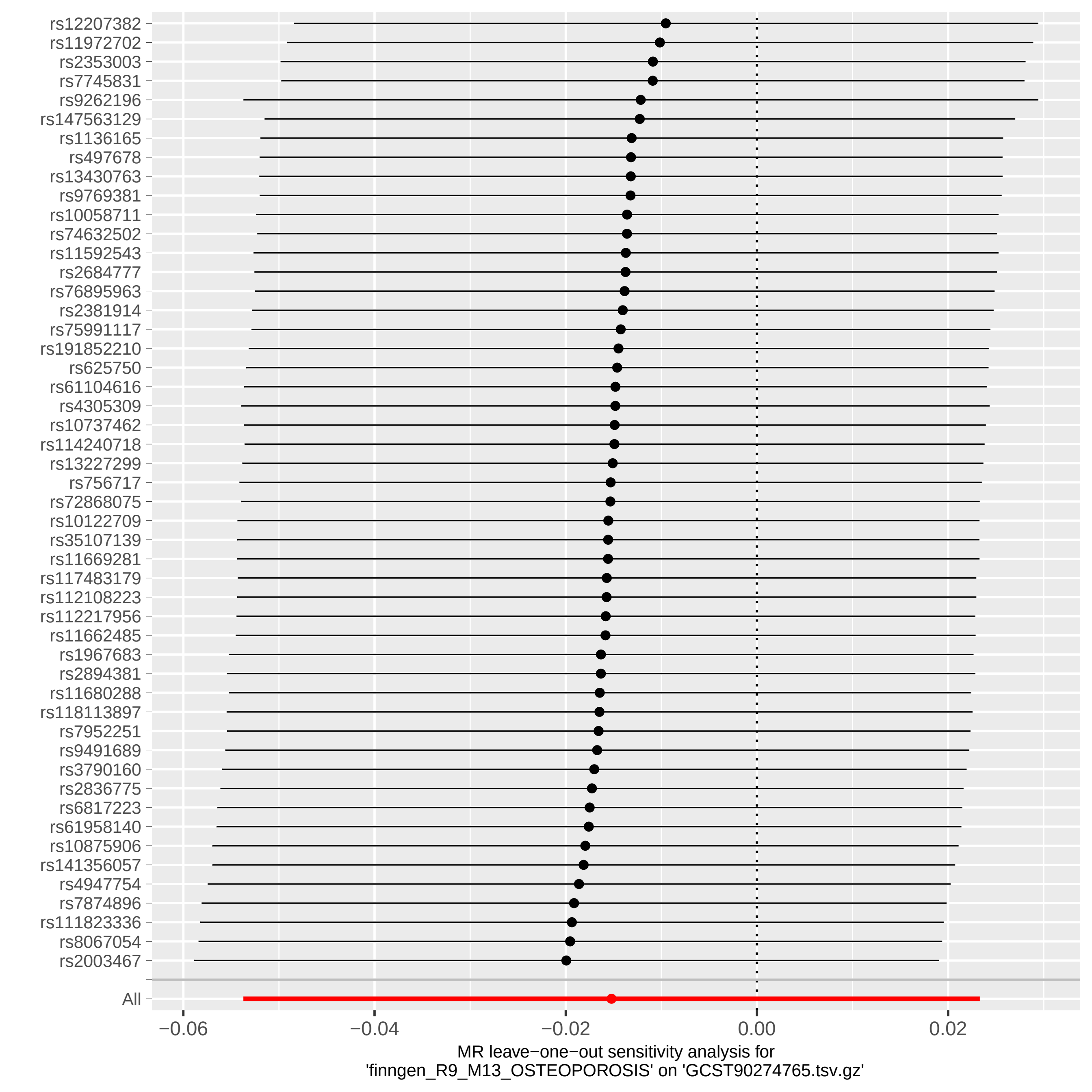
rs12207382
rs11972702
rs2353003
rs7745831
rs9262196
rs147563129
rs1136165
rs497678
rs13430763
rs9769381
rs10058711
rs74632502
rs11592543
rs2684777
rs76895963
rs2381914
rs75991117
rs191852210
rs625750
rs61104616
rs4305309
rs10737462
rs114240718
rs13227299
rs756717
rs72868075
rs10122709
rs35107139
rs11669281
rs117483179
rs112108223
rs112217956
rs11662485
rs1967683
rs2894381
rs11680288
rs118113897
rs7952251
rs9491689
rs3790160
rs2836775
rs6817223
rs61958140
rs10875906
rs141356057
rs4947754
rs7874896
rs111823336
rs8067054
rs2003467
All
−0.06
−0.04
−0.02
0.00
0.02
MR leave−one−out sensitivity analysis for
'finngen_R9_M13_OSTEOPOROSIS' on 'GCST90274765.tsv.gz'
